# Rural–urban disparities in diabetes quality of care with accountable care organization participation

**DOI:** 10.1111/jrh.70121

**Published:** 2026-02-02

**Authors:** Mariétou H. Ouayogodé, Xiyuan Hu

**Affiliations:** ^1^ Department of Population Health Sciences School of Medicine and Public Health University of Wisconsin‐Madison Madison Wisconsin USA

## Abstract

**Purpose:**

To evaluate rural–urban disparities over time in the association of ACO participation and diabetes‐related quality measures among health clinics.

**Methods:**

We used data from the Wisconsin Collaborative for Healthcare Quality all‐patient all‐payer electronic health records data system between 2011 and 2018, for patients 18–75 years. Difference‐in‐differences regression models estimated the association between ACO participation and eight diabetes quality measures among populations in rural and urban areas, separately. Triple‐difference models were also estimated to assess urban–rural disparities.

**Findings:**

Considering the two measures used in ACO performance evaluation, patients in ACO clinics were less likely to receive tobacco cessation advice relative to those in non‐ACO clinics (rural: marginal effect estimate (MEE) = –0.025, *p* = 0.033; urban: MEE = –0.231, *p* < 0.001). The triple difference across rurality was not statistically significant (MEE = –0.007 *p* = 0.56). For the remaining six ACO‐non‐incentivized measures, rural patients at ACO clinics performed better relative to their non‐ACO counterparts on kidney function monitored, and diabetes all‐or‐none optimal testing and control.

**Conclusions:**

ACO participation appeared to be more favorable for rural versus urban patients with diabetes. ACOs have potential to contribute to reducing existing rural–urban disparities in diabetes process measures.

## INTRODUCTION

Diabetes is a complex chronic condition prevalent among 38.4 million people (∼12% of adults) and affecting nearly 25% of older adults (≥65 years) in the United States.^1^, ^2^ Diabetes poor management can lead to serious disabling complications and death, making it the seventh leading cause of death in the country.^2^ Moreover, there are large geographic disparities in diabetes prevalence and outcomes.^3^, ^4^, ^5^, ^6^, ^7^ Diabetes disproportionately affects rural relative to urban communities with 17% higher prevalence in rural than in urban areas.^1^ Relative to urban residents, rural residents lack insurance at a higher rate and are more likely to be older and die prematurely from chronic diseases because rural communities have relatively fewer resources to prevent and treat chronic diseases.^8^, ^9^ Because people with diabetes often have other co‐occurring conditions (e.g., hypertension, cardiovascular diseases) and adults from non‐urban areas are disproportionately influenced by diabetes complications,^10^ ongoing monitoring through laboratory testing, disease management and care coordination across health providers are critical.^11^, ^12^, ^13^, ^14^ Medicare accountable care organizations (ACOs), a collection of health care providers which are financially incentivized for managing and coordinating care delivered to their patients including people with complex needs, may offer an opportunity for improving diabetes quality of care (see Appendix for detail on definition and incentives).

Earlier ACO research suggested that ACOs with disadvantaged patients may struggle in their performance on quality measures.^15^ Prior research has also shown uneven formation of ACOs across the country, with rural areas lagging relative to urban areas in ACO establishment.^16^ Studies have also shown modest improvements in diabetes‐related outcomes in early years of ACO participation.^17^, ^18^, ^19^ However, less is known about differences in performance on (financially incentivized vs. non‐incentivized) diabetes‐related quality measures among ACOs in rural versus urban areas compared with non‐ACO participants.

Additionally, previous research suggested that estimated relationships might differ with ACO participation^18^ and might be instable when single ACO effect rather than separate cohort effects are being reported.^20^ Nonetheless, there remains a need for assessing the potential dynamic relationship across different ACO cohorts and geographical areas over time.

This study addressed these gaps by evaluating rural–urban disparities, over time, in the association of ACO participation, among health clinics, and validated diabetes‐related quality measures, both financially incentivized and non‐incentivized by the ACO program. We hypothesized that emphasizing selected quality measures for ACO evaluation may lead to improvements in some areas (incentivized measures) and not in others (non‐incentivized measures) within ACOs. Additionally, we anticipate that improvements in quality measures will be more likely in patients getting care at ACO clinics in urban areas, where more resources are deployed versus at rural ACO clinics.

## STUDY DATA AND METHODS

This study was approved by the University of Wisconsin‐Madison Institutional Review Board.

### Data source

We leveraged data from the Wisconsin Collaborative for Healthcare Quality (WCHQ) all‐patient all‐payer electronic health records (EHR) data system between 2011 and 2018, for patients 18 to 75 years (see Appendix for additional detail on the WCHQ). Patients are attributed to health system‐clinic pairs based on their diabetes care pattern in the previous two consecutive years (i.e. the Diabetes Care Home attribution method).^21^ We identified Medicare ACOs using public sources (the Centers for Medicare and Medicaid Services) and included an indicator to determine whether the parent health system of a clinic had ever participated in an ACO program during our study period. Patients with diabetes were identified based on a recorded diagnosis in all Occupational and Environmental Medicine diagnoses (OEM) diagnoses with history. We also leveraged health‐system aggregated data publicly available from the WCHQ website,^22^ through 2024 for trend comparison analyses.

The data included 14 reporting periods between 2011 and 2018 with the reporting periods initially lasting 1 year between 2011 and 2012 and 6 months subsequently (2013–2018). The analytic samples varied across quality measures and geographical areas: 18,970 observations (5550 individuals) to 152,335 observations (34,381 individuals) for the rural subsample and 93,956 observations (25,774 individuals) to 723,190 observations (134,451 individuals) for the urban subsample (Figure S1). This study adhered to the Strengthening the Reporting of Observational Studies in Epidemiology (STROBE) reporting guideline for cohort studies.

### Measures

We included 8 quality measures as outcome measures. Two of these, notably “hemoglobin A1c (HbA1c) poor control (HbA1c>9%)” and “tobacco user receiving tobacco cessation advice,” were consistently incentivized by the ACO program during our study period. The remaining six measures were not explicitly or consistently incentivized during the study period, including “blood sugar (A1c) testing,” “blood sugar (A1c) control (HbA1c<8%),” “blood pressure control (BP<140/90),” “kidney function monitored,” “diabetes all‐or‐none process measure (optimal testing),” and “diabetes all‐or‐none outcome measure (optimal control)” (see Appendix for detailed information about ACO incentives and measure definitions in Table S1). We recognize that poor blood sugar control in the incentivized measures and blood sugar control in the non‐incentivized measures are, by definition, correlated with HbA1c testing in the non‐incentivized measures. All quality measures were defined as binary variables at the patient level, for achieving or not the criteria for the quality measures in the period of interest. A value of 1 (vs. 0) in all but the diabetes HbA1c poor control measure was considered a “positive” outcome for discussion in this study.

We included two other dichotomous variables, one indicating the post‐ACO implementation period (  = 1 if the patient's attributed clinic was affiliated with a health system that participated in Medicare ACO programs in period from 2011 to 2018, 0 otherwise) and another identifying Medicare ACO participation (  = 1 if the patient's attributed clinic was affiliated with a health system that ever participated in Medicare ACO programs, 0 otherwise). We referred to clinics affiliated with ACOs as ACO participants as well in this study.

The dummy variable indicating rurality of individual residence ( = 1 if rural, 0 urban) was derived from six rurality categories, based on a validated rural–urban geo‐disparity model constructed for Wisconsin.^23^ This model defines six rural and urban categories: rural, rural advantaged, and rural underserved, urban, urban advantaged, and urban underserved. People from rural, rural advantaged, and rural underserved areas were classified as “rural” and otherwise as “urban.” Whenever the patient was missing a residential address in the data (4% patients), we imputed rurality classification based on their clinic's location. Individual‐level self‐reported socio‐demographic covariates included gender (male or female), race/ethnicity (White, Black, Hispanic/Latino, Asian/pacific islander, American Indian/Alaska native, or other/unknown), age group (<65, 65–69, 70–75), health insurance type (commercial, Medicaid, Medicare, uninsured, or unknown), and clinical comorbidities (number of average hierarchical condition categories (HCCs)) measured during the reporting period.

### Statistical analysis

The analysis was done at the patient‐report level. To ensure comparability of non‐ACO and ACO health systems (and their clinics), we restricted the sample to (1) large health systems with average number of patients larger than 1000 and (2) non‐ACO clinics operating within the same hospital referral regions (HRRs) as Medicare ACO‐affiliated clinics. HRRs are usually used to define local/regional health care markets. Socio‐demographic characteristics of patients were compared across rurality for ACO‐affiliated clinics and non‐ACO clinics. We estimated difference‐in‐differences (DID) with population average via generalized estimating equations (GEE) regression models with exchangeable correlation structure and logit link function to evaluate the association between ACO participation and diabetes quality measures among populations, separately for rural and urban areas. The regression models took the general form:

(1) $$\begin{array}{ccc} \log\left(\frac{Y_{ict}}{1-Y_{ict}}\right) & = & \eta +\beta \left(Post\_{ACO}_{ct}\right)+\alpha {ACO}_{c}+\theta X_{ict}+\tau_{t}\\ & & +\;\varepsilon_{ict},Y_{ict}∼Bernoulli\left(Y_{ict}\right), \end{array}$$

where $Y_{ict}$ refers to the quality measure of interest for patient i attributed to clinic c in year t. $Post\_ACO_{ct}$ is a dummy variable, taking the value of 1 for a patient's clinic affiliated with a health system ever participating in a Medicare ACO program post‐participation in reporting period t, and 0 otherwise. This is similar to an interaction term between a post‐participation period indicator and an ACO participation indicator in a standard DID. Our coefficient of interest is the coefficient corresponding to the interaction term, β, measuring the change in the population average quality measure, after adjusting for covariates and accounting for correlation between patient experiences within clinics. $ACO_{c}$ is an indicator for whether the patient's clinic, c, was affiliated with a Medicare ACO health system or not. $X_{ict}$ is a vector of individual‐level covariates (gender, race, age group, insurance type, and number of HCCs). With the reporting period representing the unit of time used in the analysis, $\tau_{t}$ denotes time fixed effects and adjusts for period‐specific factors. $\varepsilon_{ict}$ is an error term. The regression models were estimated separately by rurality. Furthermore, we assessed disparities across rurality with a triple difference regression model, adding an indicator for the patient's rural/urban residence and interaction terms between $ACO_{c}$ and $urban_{ict}$, and between $Post\_ACO_{ct}$ and $urban_{ict}$.

We also performed heterogeneity analyses across the six rural categories in the rural–urban geo‐disparity model. We then conducted a series of sensitivity analyses to assess the robustness of the main results. In these analyses we re‐estimated our main regression models including additional comorbidity measures, restricting the sample to patients aged ≥65 years, and restricting sample to only 1‐year reporting periods. For the blood sugar outcome variables, we restricted the range of values to 4%–15% to align with testing device ranges. For the tobacco measure, previously restricted to diabetes patients, we broadened the definition of that measure to include non‐diabetic patients as well and re‐estimated the regression model.

Heteroscedasticity‐robust standard errors were estimated for all models. Statistical tests were two tailed, with a *p*‐value < 0.05 considered significant. All analyses were performed using STATA, version 18.0/SE (StataCorp).

## RESULTS

### Main analysis

In rural areas, more patients in ACO clinics were male (56.58% vs. 53.41% in non‐ACO clinics, *p* = 0.004), non‐White (51.41% vs. 14.70%, *p* < 0.001), younger adults (68.51% vs. 64.05% for the <65 age group, 16.16% vs. 18.47% for 65‐ to 69‐year‐olds, 15.33% vs. 17.48% for 70‐ to 75‐year‐olds, *p* < 0.001), had private health insurance (61.00% vs. 22.58%, *p* < 0.001), and had less comorbidities as measured by the number of HCCs (4.02 vs. 5.16, *p* < 0.001). In urban areas however, patients in ACO clinics had a higher proportion of White patients (65.34% vs. 61.53% in non‐ACO clinics, *p* < 0.001) (Table 1).

**TABLE 1:** Descriptive characteristics of patients at baseline (2011–2012).

Variables	Rural			Urban		
	ACO	Non‐ACO	*p*‐value	ACO	Non‐ACO	*p*‐value
	(*N* = 5631)	(*N* = 3157)		(*N* = 19,385)	(*N* = 20,199)	
	*N* (%)	*N* (%)		*N* (%)	*N* (%)	
*Gender*						
Male	3186 (56.58)	1686 (53.41)	0.004	10,310 (53.19)	9614 (47.61)	<0.001
Female	2445 (43.42)	1471 (46.59)		9075 (46.81)	10,580 (52.39)	
*Race*						
White	2736 (48.59)	2693 (85.30)	<0.001	12,667 (65.34)	12,429 (61.53)	<0.001
Black	21 (0.37)	24 (0.76)		394 (2.03)	4989 (24.70)	
Hispanic/Latino	229 (4.07)	31 (0.98)		823 (4.25)	1184 (5.86)	<0.001
Asian/Pacific Islander	11 (0.20)	4 (0.13)		244 (1.26)	226 (1.12)	
American Indian/Alaska Native	13 (0.23)	6 (0.19)		49 (0.25)	51 (0.25)	
Other/unknown	2621 (46.55)	399 (12.64)		5208 (26.87)	1320 (6.53)	
*Age group*						
<65	3858 (68.51)	2022 (64.05)	<0.001	14,009 (72.27)	14,028 (69.45)	<0.001
65–69	910 (16.16)	583 (18.47)		2963 (15.29)	3239 (16.04)	
70–75	863 (15.33)	552 (17.48)		2413 (12.45)	2932 (14.52)	
*Insurance type*						
Commercial health insurance	3435 (61.00)	713 (22.58)	<0.001	9942 (51.29)	5248 (25.98)	<0.001
Medicaid	314 (5.58)	172 (5.45)		1418 (7.31)	1341 (6.64)	
Medicare	1441 (25.59)	1060 (33.58)		5054 (26.07)	5400 (26.73)	
Uninsured	435 (7.73)	137 (4.34)		2929 (15.11)	499 (2.47)	
Unknown	6 (0.11)	1075 (34.05)		42 (0.22)	7711 (38.18)	
Number of HCCs, mean (std.) dev)	4.02 (3.73)	5.16 (4.68)	<0.001	4.35 (4.08)	5.31 (4.91)	<0.001

*Note*: Poisson regression was used to test differences between ACO and non‐ACO groups for continuous variables and Pearson test was used for binary (proportions) variables. Most individuals aged 65 and older are typically enrolled in Medicare Fee‐for‐Service (FFS) or Medicaid FFS. Some individuals under 65, who may not qualify for Medicare or Medicaid FFS due to factors such as unemployment or immigration status, are instead covered by Medicare Health Maintenance Organization (HMO) or Medicaid HMO plans. Commercial health insurance in this dataset includes employer‐sponsored insurance, Medicare HMO, and Medicaid HMO. Inference. *p < 0.05, **p < 0.01, ***p < 0.001.

Abbreviations: ACO, accountable care organization; HCC, hierarchical condition category.

Since ACO is implemented at the health system level, the parallel trend assumption of the DID would need to be examined between health systems participating in ACOs and non‐ACO participating health systems using aggregated individual data. Although we had only 1 year of data pre‐2012, the unadjusted trends in health systems across quality measures showed some pre‐event parallel trends across ACO and non‐ACO groups (Figure S2). Additionally, the quality measures from 2018 to 2024 exhibited similar trends to those observed prior to 2018 (Figure S3). The observed differences may be attributed to variations in data availability across health systems and specific quality measures.

**FIGURE 1:**
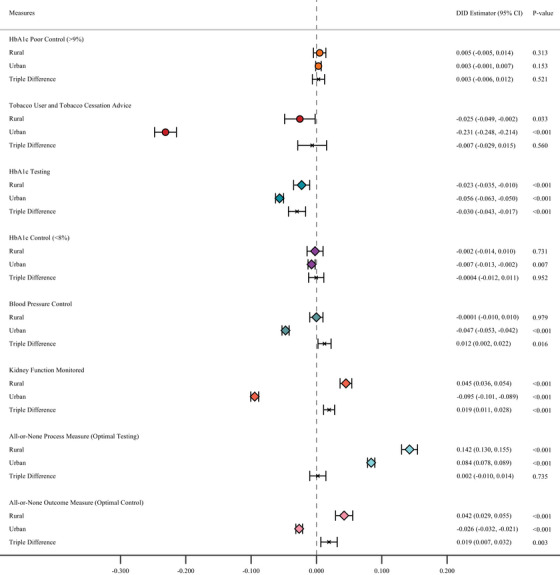
DID estimates of the association of ACO participation and ACO measures by rurality. Circles represent ACO‐incentivized measures and diamonds represent non‐incentivized measures. Generalized estimating equations (GEE) with logit link function were estimated with population averages for the association between clinic ACO affiliation and changes in the likelihood of the outcome variables, estimates of marginal effects for the interaction term between the ACO contract time and ACO participation indicators and their corresponding 95% confidence intervals were reported. All models were adjusted for factors involving gender, race, age group, insurance type, and number of HCCs. Heteroskedasticity robust standard errors were estimated. ACO, accountable care organization; DID, difference in differences.

We first assessed heterogeneity in diabetes quality of care with ACO participation across rural–urban gradients (Figure 1 and Table S2). For ACO incentivized measures, there were no statistically significant differences in HbA1c poor control among patients attributed to ACO and non‐ACO clinics in both rural and urban areas. Patients served by ACO clinics were less likely to receive tobacco cessation advice relative to those attributed to non‐ACO clinics in both groups (rural: marginal effect estimate (MEE) = –0.025, *p* = 0.033; urban: MEE = –0.231, *p* < 0.001). The triple difference across rurality was not statistically significant (MEE = ‐0.007, *p* = 0.560).

We next considered non‐incentivized measures. Patients attributed to ACO clinics in urban areas were less likely to attain both blood sugar control and blood pressure control compared to their non‐ACO counterparts), while no significant differences were found among patients living in rural areas. Additionally, there was a statistically significant difference across urbanicity in the triple difference model for blood pressure control (MEE = 0.012, *p* = 0.016). Nonetheless, patients attributed to ACO clinics in both rural and urban areas were less likely to get a blood sugar test compared to those attributed to non‐ACO clinics, and the estimated difference among rural patients was relatively lower than for their urban counterparts (MEE for triple difference = –0.030, *p* < 0.001). Although, ACO patients in urban areas were less likely than non‐ACO urban patients to get their kidney function monitored (MEE = –0.095, *p* < 0.001) and diabetes all‐or‐none optimal control (MEE = –0.026, *p* < 0.001) relative to their non‐ACO counterparts, ACO patients in rural areas outperformed rural non‐ACO patients on the two metrics (MEE = 0.045, *p* < 0.001; MEE = 0.042, *p* < 0.001, respectively). The differences across rural and urban groups were statistically significant (MEE for triple difference = 0.019, *p* < 0.001; MEE for triple difference = 0.019, *p* = 0.003, respectively). Results on the diabetes all‐or‐non optimal testing suggested that ACO patients outperformed non‐ACO patients in both rural and urban areas, but with no statistically significant difference across urbanicity (MEE for triple difference = 0.002, *p* = 0.735).

### Heterogeneity analysis

Given the more disaggregated classification and prior evidence of disparities within rural and urban areas in the geo‐disparity model, we assessed these disparities along the disaggregated gradients (Figure 2, Panels A and B and Table S3). There were still no statistically significant differences in blood sugar poor control and control measures between ACO patients and non‐ACO patients across all six rural–urban categories. The estimated decrease in performance on tobacco cessation advice estimated among ACO patients compared to non‐ACO patients in urban areas was primarily driven by patients living in urban‐advantaged areas, whereas worse performance on blood sugar testing was largely driven by outcomes of patients residing in urban and urban‐underserved areas.

FIGURE 2Heterogeneity analyses by the Wisconsin geo‐disparity model rurality categories. Circles represent ACO‐incentivized measures and diamonds represent non‐incentivized measures. Generalized estimating equations (GEE) with logit link function were estimated with population averages for the association between clinic ACO affiliation and changes in the likelihood of the outcome variables, estimates of marginal effects for the interaction term between the ACO contract time and ACO participation indicators and their corresponding 95% confidence intervals were reported. All models were adjusted for factors involving gender, race, age group, insurance type, and number of HCCs. Heteroskedasticity robust standard errors were estimated. Due to the small sample size of the rural underserved population, we combined the rural and rural underserved groups into a category. Because of this, the two categories “rural” and “rural or rural underserved” are not mutually exclusive. ACO, accountable care organization; DID, difference in differences.

**Figure:**
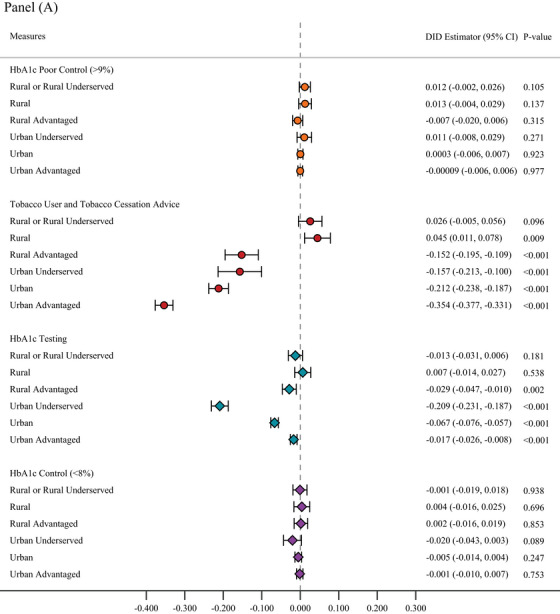

**Figure:**
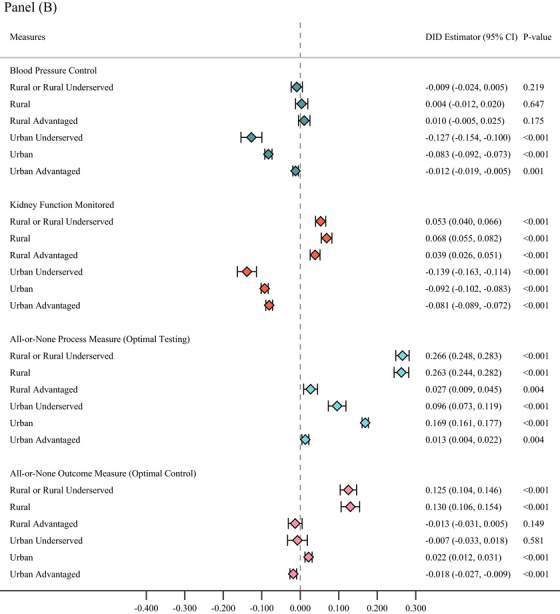

ACO patients consistently outperformed non‐ACO patients in getting their kidney function monitored across all three rural geographical area types, while the opposite was true for urban ACO patients. The relatively lower performance on blood pressure control for ACO patients in urban areas was driven primarily by those living in urban underserved and urban areas. In contrast, the higher performance estimated among rural ACO patients compared to rural non‐ACO patients on diabetes all‐or‐none optimal testing and optimal control were driven by patients in rural and rural underserved areas.

### Robustness checks

We conducted a series of analyses to assess robustness checks or our main findings. First, we included four additional HCC variables to account for the potential confounding of comorbidities and the estimates were robust to these analyses (Table S4, Panel B). Second, we carried out the analyses restricting the sample to people aged 65 years and older, and the results remained consistent in general, except for estimates of blood sugar testing in rural areas and blood sugar control in urban areas that reduced in magnitude and statistical significance (Table S4, Panel C). Third, we conducted analysis among 1‐year reporting periods, the results remained unchanged except for estimates of blood sugar poor control (gained magnitude and statistical significance) and all‐or‐none process measure (optimal testing, flipped signs) in urban areas (Table S4, Panel D). Fourth, after restricting the range of blood sugar test values 4%–15% for measures of diabetes blood sugar poor control and blood sugar control, results did not change (Table S4, Panel E). Fifth, we removed the restriction to patients with a diabetes diagnosis for the tobacco measure and results remained statistically significant as in the main analyses (Table S4, Panel F).

## DISCUSSIONS

To the best of our knowledge, this is the first study to assess the association of ACO participation and rural–urban disparities in diabetes quality of care. The association between ACO participation and diabetes‐related outcomes differed in rural and urban areas. Overall, ACO participation appeared to be associated with better outcomes for rural relative to urban patients (and clinics) on kidney function monitored and diabetes all‐or‐none optimal control.

Previous research suggested that collaboration is necessary between rural ACO providers and urban health care systems for better performance.^24^ Mixed evidence was found in the literature as for the health disparities across rurality in the relationships between ACO participation and process quality measures. Another study found that although rural–urban categories were not statistically significantly related to quality performance in 2014, ACOs serving urban counties had significantly better performance than ACOs serving counties that were mostly urban or mixed in 2015 and these ACOs serving urban counties performed better than those targeting rural county populations.^25^ The same study also showed evidence for a large variation in rural ACOs’ performance.^25^ Other research documented that ACO affiliation was not significantly associated with diabetes‐related hospitalization rates among rural older adults.^26^ Unlike these earlier studies, our results, based on a much longer time frame than previous studies, showed improved diabetes quality measures in rural areas and suggest that ACO participation could contribute to reducing existing disparities in diabetes process measures across rural and urban areas.

Most notably, our findings showed differential higher levels of certain quality measures, but not others, associated with ACO participation, consistent with findings from previous longitudinal estimates.^18^ However, some of the higher levels estimated in non‐incentivized quality measures were more notable in rural versus urban areas. ACOs are designed to aim at achieving cost containment in health care delivery while enhancing the overall quality of patient care.^27^ The former was largely achieved, as prior studies have shown that ACO participation led to reductions in health care spending,^18^, ^28^ with these reductions increasing over time.^28^ In contrast, the latter reflects a more complex and nuanced outcome as shown in our study and previous research.^17^, ^18^, ^19^ Measuring quality performance presents greater challenges than assessing health care costs, as it encompasses diverse dimensions of care quality, staff engagement, and clinical processes. As such, differential outcomes may stem from the inherent nature of specific quality measures, which can influence both performance incentives and the precision of measurement. However, our finding that diabetes patients residing in rural areas had relatively higher levels of certain quality measures, than their urban counterparts, from ACO participation, suggests that ACOs may be contributing to an implicit third aim: the reduction of health disparities for certain conditions.

## LIMITATIONS

Some limitations are worth noting. First, our study used data from the state of Wisconsin, which may limit the generalizability of results to other states. Nevertheless, this study provides valuable insights into changes in rural–urban disparities in quality performance associated with Medicare ACO participation. Second, while we examined rural–urban differences in diabetes quality of care after ACO participation, future research may want to explore how these differences interact with both taxonomy of ACOs like size and service scope and intersectional characteristics of beneficiaries such as race/ethnicity and gender. Third, our primary analysis covered the period from 2011 to 2018. Although the impact of ACO participation may have evolved since then, our extended analysis of aggregated data up to 2024 showed trends in the quality measures that were consistent with those from our main study period. It is worth noting that while quality measure data during the COVID‐19 pandemic were not publicly available on WCHQ, prior evidence using national data suggests that overall quality performance, as measured by overall quality scores, remained resilient despite a temporary decline in 2020,^29^ while worsened performance on diabetes and blood pressure control were also observed.^30^ Fourth, although the attribution algorithms for different ACO programs may impact the characteristics of the study population,^31^ patient attribution in this study is based on a different diabetes‐specific attribution: the diabetes care home attribution method. This attribution is based on the patients’ diabetes care pattern in the previous two consecutive years and is used across all health systems (ACO and non‐ACO participants). All patients were included and were not restricted to Traditional (fee‐for‐service) Medicare patients and analyses restricted to patients ≥65 years produced similar findings. Related to this limitation, we are unable to distinguish between Medicare advantage, Program of All‐inclusive Care for the Elderly (PACE), and fee‐for‐service Medicare patients. Fifth, the quality measures were defined at the reporting period rather than the patient visit level. And we could not examine differences in patient visits per reporting period that could contribute to differential outcomes across rural versus urban ACO practice settings. Sixth, although regressions adjusted for the patient's average HCCs during the reporting period, we did not have data on more specific patient health risk factors to include in the analyses.

## PUBLIC HEALTH IMPLICATIONS

Our findings can have important public health implications regarding bridging health care quality disparities across rural and urban areas. Although ACO programs were not designed explicitly for reducing health care disparities across rurality, our findings provide insights about the potential role of ACO programs in reducing disparities in overall quality performance and diabetes quality of care, across rural and urban areas.

The study also invites policymakers to consider developing quality measures relevant to rural–urban geographical gradients and providing the necessary financial and resource support to rural organizations and communities. There is a need for establishing policies that are effective in lowering risk of diabetes, addressing obesity epidemic, and in improving overall quality of diabetes care as well as access to diabetes management in rural areas.  Some strategies for improving diabetes management in rural areas have been implemented (e.g., telemedicine)^32^, ^33^ but can be reinforced with additional resources (e.g., diabetes self‐management education and support, DSMES) to such communities. Future studies might probe for additional interventions aimed at improving access to care across geographies and reducing barriers to health care services in rural communities.

## CONFLICT OF INTEREST STATEMENT

The authors have no conflicts of interest to report.

## DISCLOSURES

The authors have no disclosures to report.

## Supporting information



Supporting Information
