# Supplementary material for: Rural–urban disparities in diabetes quality of care with accountable care organization participation
Source: J Rural Health. 2026 Feb 2;42(1):e70121. doi: 10.1111/jrh.70121 (PMC12863121; doi:10.1111/jrh.70121)
Supplement: Supplementary file 1 — Supporting Information [file JRH-42-e70121-s001.docx]

# Supplementary Appendix

*The Wisconsin Collaborative for Healthcare Quality data*

The WCHQ is a voluntary statewide consortium of healthcare organizations (e.g. health systems, medical groups, and hospitals) that leads the nation in measuring and reporting multiple quality and performance metrics.^1^ It is the largest aggregation of electronic health records (EHR) patient- and encounter-level data from non-academic healthcare systems in the country. It provides all-patient all-payer EHR data for patients ≥18 years. Since 2003, the WCHQ has grown to 35 healthcare system members that publicly report diabetes quality measures. Members represent 65% of Wisconsin’s primary care providers and a wide variety of clinical settings from community-based practices to large, integrated and academic health systems that serve rural and urban communities.^2^ An exclusive gain to partnering with the WCHQ is that members agree on common quality metrics, including using the same definitions for the numerator and denominator populations, and publicly report their performance. This study used the WCHQ data between 2011 and 2018, for patients 18 to 75 years

We used 8 quality measures: hemoglobin A1c (HbA1c) poor control (HbA1c>9%), tobacco user receiving tobacco cessation advice, blood sugar (A1c) testing, blood sugar (A1c) control (HbA1c<8%), blood pressure control (BP<140/90), kidney function monitored, diabetes all-or-none process measure (optimal testing), and diabetes all-or-none outcome measure (optimal control). The patient’s HbA1c testing value served to identify whether their HbA1c was in poor control or not. The tobacco user receiving tobacco cessation advice was determined by self-reported data on tobacco use and cessation advice.

For each quality metric and reporting period, the collaborative members (i.e. health systems) submit data on their individual clinics. For each quality metric, these clinics-level data include the clinics’ attributed patients included in the metric’s denominator with additional indicator for whether the patient was also included in the numerator (met the metric’s event criteria) together with the patient’s associated socio-demographic data. Sociodemographic data include gender (male or female), race/ethnicity (White, Black, Hispanic/Latino, Asian/pacific islander, American Indian/Alaska native, or other/unknown), age group (<65, 65-69, 70-75), health insurance (commercial, Medicaid, Medicare, uninsured, or unknown), and clinical comorbidities (number of average hierarchical condition categories (HCCs)) measured during the reporting period. The data also included patients’ self-reported address of residence that served to identify the rurality of their residence. The rurality variable included six rurality categories, based on a validated rural-urban geo-disparity model constructed for Wisconsin.^3^ This model defines six rural and urban categories: rural, rural advantaged, and rural underserved, urban, urban advantaged, and urban underserved. In this study people from rural, rural advantaged, and rural underserved areas were classified as “rural” and otherwise as “urban”.

For example, the clinic’s aggregated diabetes blood sugar (A1c) testing metric assesses the percentage of patients aged 18 to 75 years with a diagnosis of diabetes who had any A1c tests within the measurement period. For each diabetic patients aged 18-75 years in the clinic’s denominator, we also have information on whether or not they had an A1c testing during that reporting period.

*Accountable Care Organization Incentives*

The Centers for Medicare and Medicare Services implemented the Medicare accountable care organization (ACO) program in 2012 with the Pioneer and the Medicare Shared Savings Program (MSSP). ACOs are a collection of healthcare providers which are financially incentivized for managing and coordinating care delivered to their patients including people with complex needs, offer an opportunity for improving diabetes quality of care. Participating organizations that lower their patients’ spending below pre-determined financial benchmarks and meet quality standards are eligible to share the savings generated with Medicare. The shared savings payment rates vary according to financial risk model (one-sided risk versus two-sided risk) and ACO program (e.g. Medicare Shared Savings Program-MSSP, Pioneer, Next Generation ACOs, started in 2016). The MSSP, the largest and permanent ACO program, is the only program operating since 2021 as the other more advanced programs have ended (Pioneer in 2016 and Next Generation ACO in 2021). Medicare ACO quality measures include over 30 measures spanning domains including patient/caregiver experience, care coordination/patient safety, preventive health, and at-risk population (including diabetes, hypertension, ischemic vascular disease, heart failure, coronary artery disease, and depression). Among the at-risk population conditions targeted by the ACO program, diabetes had the highest number of individual quality measures used by the ACO program. Up to 2014, the ACO program diabetes quality measure was a composite measure including 5 different measures (ACO #22. Hemoglobin A1c Control (HbA1c) (<8 percent), ACO #23. Low Density Lipoprotein (LDL) (<100 mg/dL), ACO #24. Blood Pressure (BP) < 140/90, ACO #25. Tobacco Non Use, and ACO #26. Aspirin Use) and ACO #27.^4^ Percent of beneficiaries with diabetes whose HbA1c in poor control (>9 percent). From 2015 to 2018, the previous composite measure was no longer used but a different composite measure was defined for the performance evaluation including two measures ( ACO #27. Percent of beneficiaries with diabetes whose HbA1c in poor control (>9 percent) and ACO-41: Diabetes: Eye Exam.^5^ Since 2019 the program has used one diabetes-specific quality measure: ACO #27. Percent of beneficiaries with diabetes whose HbA1c in poor control (>9 percent).^5^

**Reference:**

1. Hatahet MA, Bowhan J, Clough EA. Wisconsin Collaborative for Healthcare Quality (WCHQ): lessons learned. *WMJ: Official Publication of the State Medical Society of Wisconsin*. 2004;103(3):45-48.

2. Wisconsin Collaborative for Healthcare Quality. 2019 Wisconsin Health Disparities Report. 2019.

3. Bonham-Werling J, DeLonay AJ, Stephenson K, et al. Using Statewide Electronic Health Record and Influenza Vaccination Data to Plan and Prioritize COVID-19 Vaccine Outreach and Communications in Wisconsin Communities. *Am J Public Health*. 2021;111(12):2111-2114. doi:10.2105/AJPH.2021.306524

4. Table: 33 ACO Quality Measures. Shared Savings Program. Centers for Medicare & Medicaid Services. Accessed 10-22-2025. https://www.cms.gov/medicare/medicare-fee-for-service-payment/sharedsavingsprogram/downloads/aco-shared-savings-program-quality-measures.pdf.

5. Performance Year Financial and Quality Results. Centers for Medicare & Medicaid Services. Accessed 10-22-2025. https://data.cms.gov/medicare-shared-savings-program/performance-year-financial-and-quality-results.

# Supplemental Figure 1. Flowchart of Sample Selection


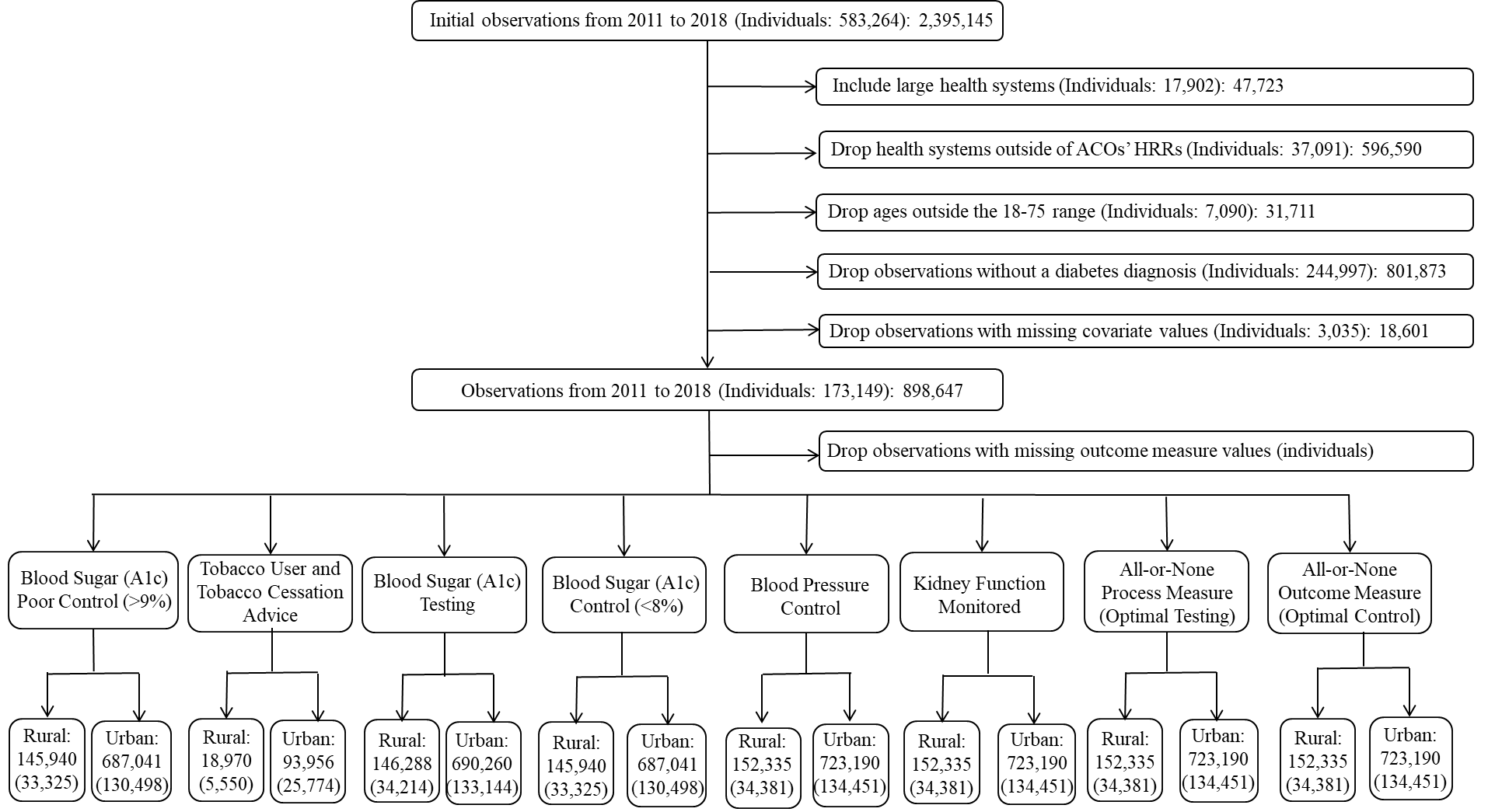


Abbreviations: HRRs: hospital referral regions.

**Notes**: Observations represent the number of patient-report observations. From 2011 to 2012, the reporting period was annual with an end date of June 30 each year. From 2013 to 2018 however, reporting periods lasted 6 months and ended on June 30 and December 31 each year. A total of 14 reporting periods with a length of either half a year or one year were included. HRRs were used to identify ACO local markets.

# Supplemental Figure 2. Unadjusted Trends in Quality Measures Over Time


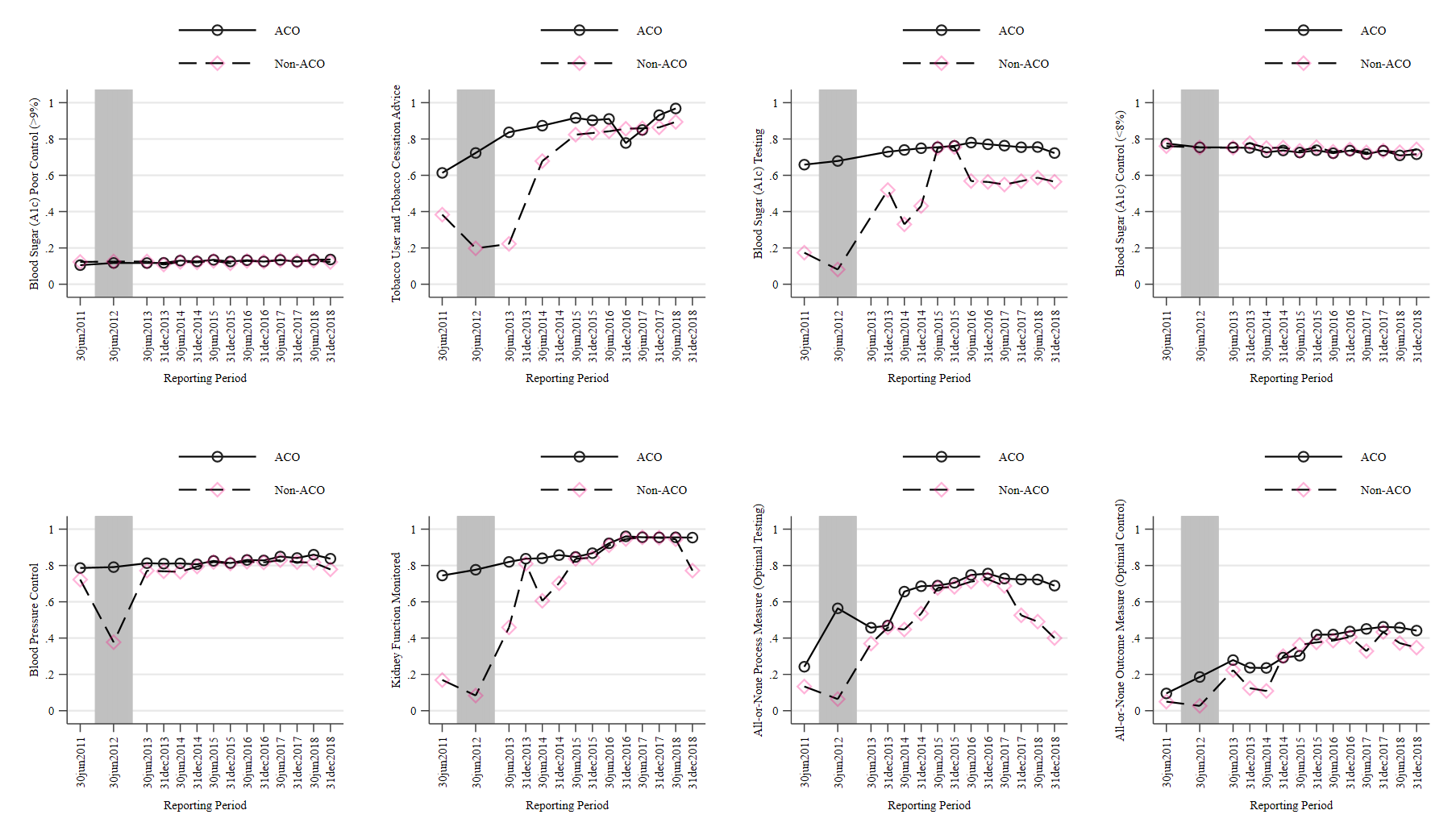


Abbreviations: ACO: accountable care organization.

# Supplemental Figure 3. Trends in Quality Measures: Comparison Between Study Data and WCHQ Website, Reporting Period From 2011 To 2024


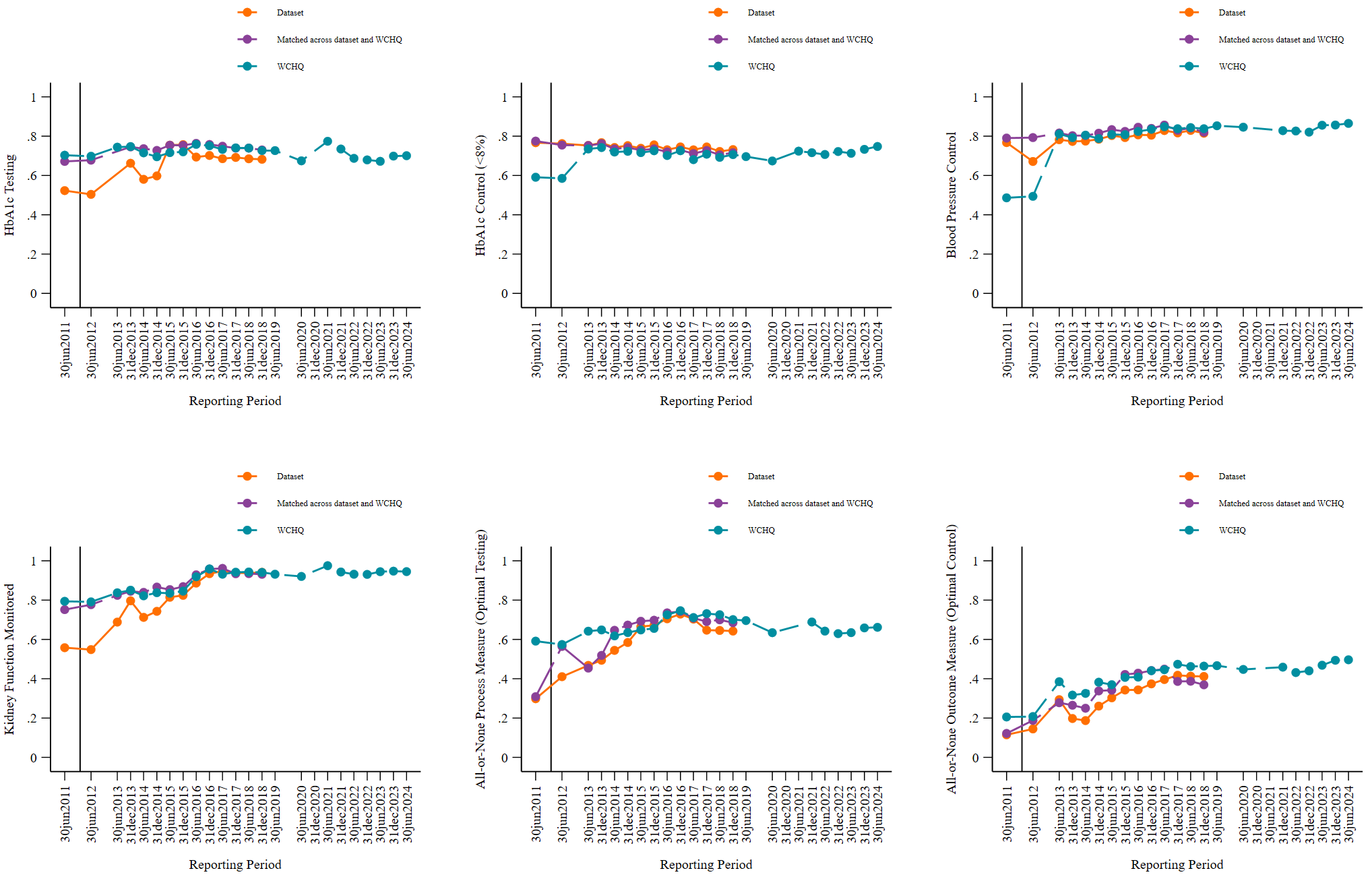


Abbreviations: ACO: accountable care organization. WCHQ: Wisconsin Collaborative for Healthcare Quality.

**Notes**: The *Dataset* label refers to the electronic health record individual level data we analyzed in this study. The *WCHQ* label on the figure refers to the publicly available data from the WCHQ website. The *Dataset* label refers to the electronic health record individual level data we analyzed in this study. The *WCHQ* label on the figure refers to the publicly available data from the WCHQ website. Orange line indicates the average number of each quality measure equaling 1 for individual level data. Blue line indicates the proportion of patients meeting criteria, which is from public available WCHQ website (<https://reports.wchq.org/measures/1>). Purple line indicates the average number of each quality measure equaling 1 for individual level data with health system matched across individual level data and WCHQ website. 6 quality measures (all non-incentivized) were available from the WCHQ website: Blood Sugar (A1c) Testing, Blood Sugar (A1c) Control (<8%), Blood Pressure Control, Kidney Function Monitored, All-or-None Process Measure (Optimal Testing), and All-or-None Outcome Measure (Optimal Control). A total of 13 health systems published their performance on the 6 quality measures on the public WCHQ website while our data included individual level data across 20 health systems on the 8 measures. Observed differences across trends and datasets were likely due in part to variations in data availability across health systems and quality measures.

# Supplemental Table 1. Definition of Quality Measures

| **Quality Measure** | **Definition** |
| --- | --- |
| ***ACO Incentivized Measures*** |  |
| Blood Sugar (A1c) Poor Control (>9%) | Person 18 to 75 years of age with a diagnosis of diabetes with a A1c level greater than 9.0%-uncontrolled A1c |
| Tobacco User and Tobacco Cessation Advice | Person 18 to 75 years of age with diabetes, a tobacco user and has received tobacco Cessation advice |
| ***Non-Incentivized Measures*** |  |
| Blood Sugar (A1c) Testing | Person 18 to 75 years of age with a diagnosis of diabetes who had two or more A1c tests, one A1c test, or no A1c tests within the measurement year. |
| Blood Sugar (A1c) Control (<8%)† | Person 18 to 75 years of age with a diagnosis of diabetes with a A1c level controlled to less than 8.0%-good control |
| Blood Pressure Control† | Person 18 to 75 years of age with a diagnosis of diabetes whose most recent blood pressure reading within the measurement period is controlled to a rate of less than 140/90 mmHg. |
| Kidney Function Monitored | Person 18 to 75 years of age with a diagnosis of diabetes who was screened and/or monitored for kidney disease in the measurement year. |
| All-or-None Process Measure (Optimal Testing) | Person with diabetes reaching all three goals for meeting the diabetes optimal testing measure including:  -Two A1c tests performed during the 12 month reporting period -- and  -One kidney function test during the 12 month reporting period, and/or diagnosis and treatment of kidney disease -- and  - One estimated Glomerular Filtration Rate (eGFR) test annually |
| All-or-None Outcome Measure (Optimal Control) | Person with diabetes reaching all five goals for meeting the diabetes optimal control outcome measure including :  -Most recent A1c test result is less than 8.0% -- and  -Most recent blood pressure measurement is less than 140/90 mm Hg -- and  -Tobacco Non-User -- and  -Daily Aspirin or Other Antiplatelet for Diabetes Patients with Ischemic Vascular Disease (IVD) Unless Contraindicated -- and  - Statin Use for patients ages 40 through 75 or patients with IVD of any age |

Notes: Adapted from measure definitions from the Wisconsin Collaborative for Healthcare Quality (<https://reports.wchq.org>)

†ACO #22 Hemoglobin A1c Control (HbA1c) (<8 %) and ACO #24. Blood Pressure (BP) < 140/90 were used in ACO performance evaluation in the first 2 performance years (2013-2014) and removed starting in 2015.

# Supplemental Table 2. DID Estimates of the Association of ACO Participation and ACO Measures Across Rurality

| Variables | Blood Sugar (A1c) Poor Control (>9%) | | | Tobacco User and Tobacco Cessation Advice | | | Blood Sugar (A1c) Testing | | | Blood Sugar (A1c) Control (<8%) | | |
| --- | --- | --- | --- | --- | --- | --- | --- | --- | --- | --- | --- | --- |
|  | (1) | (2) | (3) | (4) | (5) | (6) | (7) | (8) | (9) | (10) | (11) | (12) |
|  | Rural | Urban | Triple | Rural | Urban | Triple | Rural | Urban | Triple | Rural | Urban | Triple |
| Post | 0.005 | 0.003 | 0.003 | -0.025 | -0.231*** | -0.192*** | -0.023*** | -0.056*** | -0.046*** | -0.002 | -0.007* | -0.007* |
|  | (0.005) | (0.002) | (0.002) | (0.012) | (0.009) | (0.008) | (0.006) | (0.003) | (0.003) | (0.006) | (0.003) | (0.003) |
| Post*Rurality |  |  | 0.003 |  |  | -0.007 |  |  | -0.030*** |  |  | -0.0004 |
|  |  |  | (0.005) |  |  | (0.011) |  |  | (0.007) |  |  | (0.006) |
| ACO | Yes | Yes | Yes | Yes | Yes | Yes | Yes | Yes | Yes | Yes | Yes | Yes |
| Rurality |  |  | Yes |  |  | Yes |  |  | Yes |  |  | Yes |
| ACO* Rurality |  |  | Yes |  |  | Yes |  |  | Yes |  |  | Yes |
| Covariates | Yes | Yes | Yes | Yes | Yes | Yes | Yes | Yes | Yes | Yes | Yes | Yes |
| Time FE | Yes | Yes | Yes | Yes | Yes | Yes | Yes | Yes | Yes | Yes | Yes | Yes |
| Observations | 145,940 | 687,041 | 832,981 | 18,970 | 93,956 | 112,926 | 146,288 | 690,260 | 836,548 | 145,940 | 687,041 | 832,981 |

**Continued:**

| Variables | Blood Pressure Control | | | Kidney Function Monitored | | | All-or-None Process Measure (Optimal Testing) | | | All-or-None Outcome Measure (Optimal Control) | | |
| --- | --- | --- | --- | --- | --- | --- | --- | --- | --- | --- | --- | --- |
|  | (13) | (14) | (15) | (16) | (17) | (18) | (19) | (20) | (21) | (22) | (23) | (24) |
|  | Rural | Urban | Triple | Rural | Urban | Triple | Rural | Urban | Triple | Rural | Urban | Triple |
| Post | -0.0001 | -0.047*** | -0.041*** | 0.045*** | -0.095*** | -0.072*** | 0.142*** | 0.084*** | 0.091*** | 0.042*** | -0.026*** | -0.020*** |
|  | (0.005) | (0.003) | (0.003) | (0.005) | (0.003) | (0.003) | (0.006) | (0.003) | (0.003) | (0.007) | (0.003) | (0.003) |
| Post*Rurality |  |  | 0.012* |  |  | 0.019*** |  |  | 0.002 |  |  | 0.019** |
|  |  |  | (0.005) |  |  | (0.004) |  |  | (0.006) |  |  | (0.006) |
| ACO | Yes | Yes | Yes | Yes | Yes | Yes | Yes | Yes | Yes | Yes | Yes | Yes |
| Rurality |  |  | Yes |  |  | Yes |  |  | Yes |  |  | Yes |
| ACO* Rurality |  |  | Yes |  |  | Yes |  |  | Yes |  |  | Yes |
| Covariates | Yes | Yes | Yes | Yes | Yes | Yes | Yes | Yes | Yes | Yes | Yes | Yes |
| Time FE | Yes | Yes | Yes | Yes | Yes | Yes | Yes | Yes | Yes | Yes | Yes | Yes |
| Observations | 152,335 | 723,190 | 875,525 | 152,335 | 723,190 | 875,525 | 152,335 | 723,190 | 875,525 | 152,335 | 723,190 | 875,525 |

Abbreviations: ACO: accountable care organization. DID: difference in differences. HCC: hierarchical condition category.

**Notes**: The variable *Post* was defined to represent an interaction between ACO participation and time of implementation. Generalized estimating equations (GEE) with logit link function were estimated with population averages for the association between clinic ACO affiliation and changes in the likelihood of the outcome variables, marginal effects and standard errors were reported. All models were adjusted for factors involving gender, race, age group, insurance type, and number of HCCs. Heteroskedasticity robust standard errors were estimated. Inference. * p<0.05, ** p<0.01, *** p<0.001. Bonferroni correction was used.

# Supplemental Table 3. Heterogeneity Analyses by the Wisconsin Geo-Disparity Model Rurality Categories

| Variables | Blood Sugar (A1c) Poor Control (>9%) | | | |  |  | Tobacco User and Tobacco Cessation Advice | | | | |  |
| --- | --- | --- | --- | --- | --- | --- | --- | --- | --- | --- | --- | --- |
|  | (1) | (2) | (3) | (4) | (5) | (6) | (7) | (8) | (9) | (10) | (11) | (12) |
|  | Rural or Rural Underserved | Rural | Rural Advantaged | Urban Underserved | Urban | Urban Advantaged | Rural or Rural Underserved | Rural | Rural Advantaged | Urban Underserved | Urban | Urban Advantaged |
| Post | 0.012 | 0.013 | -0.007 | 0.011 | 0.0003 | -0.00009 | 0.026 | 0.045 | -0.152*** | -0.157*** | -0.212*** | -0.354*** |
|  | (0.007) | (0.008) | (0.007) | (0.010) | (0.003) | (0.003) | (0.016) | (0.017) | (0.022) | (0.029) | (0.013) | (0.012) |
| ACO | Yes | Yes | Yes | Yes | Yes | Yes | Yes | Yes | Yes | Yes | Yes | Yes |
| Covariates | Yes | Yes | Yes | Yes | Yes | Yes | Yes | Yes | Yes | Yes | Yes | Yes |
| Time FE | Yes | Yes | Yes | Yes | Yes | Yes | Yes | Yes | Yes | Yes | Yes | Yes |
| Observations | 75,525 | 61,797 | 70,415 | 123,388 | 377,194 | 186,459 | 11,274 | 9,043 | 7,675 | 20,534 | 53,594 | 19,828 |

**Continued:**

| Variables | Blood Sugar (A1c) Testing | | | |  |  | Blood Sugar (A1c) Control (<8%) | | |  |  |  |
| --- | --- | --- | --- | --- | --- | --- | --- | --- | --- | --- | --- | --- |
|  | (13) | (14) | (15) | (16) | (17) | (18) | (19) | (20) | (21) | (22) | (23) | (24) |
|  | Rural or Rural Underserved | Rural | Rural Advantaged | Urban Underserved | Urban | Urban Advantaged | Rural or Rural Underserved | Rural | Rural Advantaged | Urban Underserved | Urban | Urban Advantaged |
| Post | -0.013 | 0.007 | -0.029* | -0.209*** | -0.067*** | -0.017*** | -0.001 | 0.004 | 0.002 | -0.020 | -0.005 | -0.001 |
|  | (0.009) | (0.011) | (0.009) | (0.011) | (0.005) | (0.004) | (0.009) | (0.010) | (0.009) | (0.012) | (0.004) | (0.004) |
| ACO | Yes | Yes | Yes | Yes | Yes | Yes | Yes | Yes | Yes | Yes | Yes | Yes |
| Covariates | Yes | Yes | Yes | Yes | Yes | Yes | Yes | Yes | Yes | Yes | Yes | Yes |
| Time FE | Yes | Yes | Yes | Yes | Yes | Yes | Yes | Yes | Yes | Yes | Yes | Yes |
| Observations | 76,459 | 62,326 | 69,829 | 124,668 | 379,830 | 185,762 | 75,525 | 61,797 | 70,415 | 123,388 | 377,194 | 186,459 |

**Continued:**

| Variables | Blood Pressure Control | | |  |  |  | Kidney Function Monitored | | |  |  |  |
| --- | --- | --- | --- | --- | --- | --- | --- | --- | --- | --- | --- | --- |
|  | (25) | (26) | (27) | (28) | (29) | (30) | (31) | (32) | (33) | (34) | (35) | (36) |
|  | Rural or Rural Underserved | Rural | Rural Advantaged | Urban Underserved | Urban | Urban Advantaged | Rural or Rural Underserved | Rural | Rural Advantaged | Urban Underserved | Urban | Urban Advantaged |
| Post | -0.009 | 0.004 | 0.010 | -0.127*** | -0.083*** | -0.012** | 0.053*** | 0.068*** | 0.039*** | -0.139*** | -0.092*** | -0.081*** |
|  | (0.007) | (0.008) | (0.008) | (0.014) | (0.005) | (0.004) | (0.007) | (0.007) | (0.007) | (0.013) | (0.005) | (0.004) |
| ACO | Yes | Yes | Yes | Yes | Yes | Yes | Yes | Yes | Yes | Yes | Yes | Yes |
| Covariates | Yes | Yes | Yes | Yes | Yes | Yes | Yes | Yes | Yes | Yes | Yes | Yes |
| Time FE | Yes | Yes | Yes | Yes | Yes | Yes | Yes | Yes | Yes | Yes | Yes | Yes |
| Observations | 79,624 | 64,969 | 72,711 | 131,256 | 399,139 | 192,795 | 79,624 | 64,969 | 72,711 | 131,256 | 399,139 | 192,795 |

**Continued:**

| Variables | All-or-None Process Measure (Optimal Testing) | | | | |  | All-or-None Outcome Measure (Optimal Control) | | | | |  |
| --- | --- | --- | --- | --- | --- | --- | --- | --- | --- | --- | --- | --- |
|  | (37) | (38) | (39) | (40) | (41) | (42) | (43) | (44) | (45) | (46) | (47) | (48) |
|  | Rural or Rural Underserved | Rural | Rural Advantaged | Urban Underserved | Urban | Urban Advantaged | Rural or Rural Underserved | Rural | Rural Advantaged | Urban Underserved | Urban | Urban Advantaged |
| Post | 0.266*** | 0.263*** | 0.027* | 0.096*** | 0.169*** | 0.013* | 0.125*** | 0.130*** | -0.013 | -0.007 | 0.022*** | -0.018*** |
|  | (0.009) | (0.010) | (0.009) | (0.012) | (0.004) | (0.005) | (0.011) | (0.012) | (0.009) | (0.013) | (0.005) | (0.004) |
| ACO | Yes | Yes | Yes | Yes | Yes | Yes | Yes | Yes | Yes | Yes | Yes | Yes |
| Covariates | Yes | Yes | Yes | Yes | Yes | Yes | Yes | Yes | Yes | Yes | Yes | Yes |
| Time FE | Yes | Yes | Yes | Yes | Yes | Yes | Yes | Yes | Yes | Yes | Yes | Yes |
| Observations | 79,624 | 64,969 | 72,711 | 131,256 | 399,139 | 192,795 | 79,624 | 64,969 | 72,711 | 131,256 | 399,139 | 192,795 |

Abbreviations: ACO: accountable care organization. DID: difference in differences. HCC: hierarchical condition category.

**Notes**: The variable *Post* was defined to represent an interaction between ACO participation and time of implementation. Generalized estimating equations (GEE) with logit link function were estimated with population averages for the association between clinic ACO affiliation and changes in the likelihood of the outcome variables, marginal effects and standard errors. Due to the small sample size of the rural underserved population, we combined the rural and rural underserved groups into a category. Because of this, the two categories “rural” and “rural or rural underserved” are not mutually exclusive. All models were adjusted for factors involving gender, race, age group, insurance type, and number of HCCs. Heteroskedasticity robust standard errors were estimated. Inference. * p<0.05, ** p<0.01, *** p<0.001. Bonferroni correction was used.

# Supplemental Table 4. Results of Robustness Checks

| Panels | Variables | Blood Sugar (A1c) Poor Control (>9%) | | | Tobacco User and Tobacco Cessation Advice | | | Blood Sugar (A1c) Testing | | | Blood Sugar (A1c) Control (<8%) | | |
| --- | --- | --- | --- | --- | --- | --- | --- | --- | --- | --- | --- | --- | --- |
|  |  | (1) | (2) | (3) | (4) | (5) | (6) | (7) | (8) | (9) | (10) | (11) | (12) |
|  |  | Rural | Urban | Triple | Rural | Urban | Triple | Rural | Urban | Triple | Rural | Urban | Triple |
| A | Post | 0.005 | 0.003 |  | -0.025 | -0.231*** |  | -0.023*** | -0.056*** |  | -0.002 | -0.007* |  |
|  |  | (0.005) | (0.002) |  | (0.012) | (0.009) |  | (0.006) | (0.003) |  | (0.006) | (0.003) |  |
|  | Post*Rurality |  |  | 0.003 |  |  | -0.007 |  |  | -0.030*** |  |  | -0.0004 |
|  |  |  |  | (0.005) |  |  | (0.011) |  |  | (0.007) |  |  | (0.006) |
| B | Post | 0.005 | 0.002 |  | -0.025 | -0.231*** |  | -0.022*** | -0.056*** |  | -0.002 | -0.007* |  |
|  |  | (0.005) | (0.002) |  | (0.012) | (0.009) |  | (0.006) | (0.003) |  | (0.006) | (0.003) |  |
|  | Post*Rurality |  |  | 0.003 |  |  | -0.007 |  |  | -0.030*** |  |  | -0.0003 |
|  |  |  |  | (0.005) |  |  | (0.011) |  |  | (0.007) |  |  | (0.006) |
| C | Post | 0.008 | -0.004 |  | -0.017 | -0.250*** |  | -0.002 | -0.058*** |  | -0.004 | -0.003 |  |
|  |  | (0.006) | (0.002) |  | (0.026) | (0.018) |  | (0.010) | (0.006) |  | (0.009) | (0.004) |  |
|  | Post*Rurality |  |  | 0.008 |  |  | -0.017 |  |  | -0.025 |  |  | 0.004 |
|  |  |  |  | (0.006) |  |  | (0.025) |  |  | (0.012) |  |  | (0.009) |
| D | Post | 0.011 | 0.007* |  | 0.017 | -0.181*** |  | -0.038*** | -0.121*** |  | -0.005 | -0.016*** |  |
|  |  | (0.006) | (0.003) |  | (0.012) | (0.009) |  | (0.008) | (0.004) |  | (0.007) | (0.003) |  |
|  | Post*Rurality |  |  | 0.005 |  |  | 0.003 |  |  | -0.029*** |  |  | 0.001 |
|  |  |  |  | (0.006) |  |  | (0.012) |  |  | (0.008) |  |  | (0.007) |
| E | Post | 0.005 | 0.003 |  |  |  |  |  |  |  | -0.002 | -0.008** |  |
|  |  | (0.005) | (0.002) |  |  |  |  |  |  |  | (0.006) | (0.003) |  |
|  | Post*Rurality |  |  | 0.003 |  |  |  |  |  |  |  |  | 0.00006 |
|  |  |  |  | (0.005) |  |  |  |  |  |  |  |  | (0.006) |
| F | Post |  |  |  | -0.024*** | -0.213*** |  |  |  |  |  |  |  |
|  |  |  |  |  | (0.004) | (0.003) |  |  |  |  |  |  |  |
|  | Post*Rurality |  |  |  |  |  | 0.004 |  |  |  |  |  |  |
|  |  |  |  |  |  |  | (0.004) |  |  |  |  |  |  |

**Continued:**

| Panels | Variables | Blood Pressure Control | | | Kidney Function Monitored | | | All-or-None Process Measure (Optimal Testing) | | | All-or-None Outcome Measure (Optimal Control) | | |
| --- | --- | --- | --- | --- | --- | --- | --- | --- | --- | --- | --- | --- | --- |
|  |  | (13) | (14) | (15) | (16) | (17) | (18) | (19) | (20) | (21) | (22) | (23) | (24) |
|  |  | Rural | Urban | Triple | Rural | Urban | Triple | Rural | Urban | Triple | Rural | Urban | Triple |
| A | Post | -0.0001 | -0.047*** |  | 0.045*** | -0.095*** |  | 0.142*** | 0.084*** |  | 0.042*** | -0.026*** |  |
|  |  | (0.005) | (0.003) |  | (0.005) | (0.003) |  | (0.006) | (0.003) |  | (0.007) | (0.003) |  |
|  | Post*Rurality |  |  | 0.012* |  |  | 0.019*** |  |  | 0.002 |  |  | 0.019** |
|  |  |  |  | (0.005) |  |  | (0.004) |  |  | (0.006) |  |  | (0.006) |
| B | Post | -0.0004 | -0.048*** |  | 0.045*** | -0.094*** |  | 0.142*** | 0.084*** |  | 0.042*** | -0.026*** |  |
|  |  | (0.005) | (0.003) |  | (0.005) | (0.003) |  | (0.006) | (0.003) |  | (0.007) | (0.003) |  |
|  | Post*Rurality |  |  | 0.012* |  |  | 0.019*** |  |  | 0.002 |  |  | 0.019** |
|  |  |  |  | (0.005) |  |  | (0.004) |  |  | (0.006) |  |  | (0.006) |
| C | Post | 0.000 | -0.038*** |  | 0.062*** | -0.061*** |  | 0.160*** | 0.091*** |  | 0.046*** | -0.039*** |  |
|  |  | (0.009) | (0.005) |  | (0.007) | (0.005) |  | (0.009) | (0.005) |  | (0.012) | (0.005) |  |
|  | Post*Rurality |  |  | 0.008 |  |  | 0.015 |  |  | 0.002 |  |  | 0.026* |
|  |  |  |  | (0.009) |  |  | (0.007) |  |  | (0.011) |  |  | (0.011) |
| D | Post | -0.002 | -0.083*** |  | -0.018** | -0.147*** |  | 0.061*** | -0.027*** |  | 0.018* | -0.048*** |  |
|  |  | (0.006) | (0.004) |  | (0.006) | (0.004) |  | (0.007) | (0.003) |  | (0.007) | (0.003) |  |
|  | Post*Rurality |  |  | 0.023*** |  |  | 0.029*** |  |  | -0.005 |  |  | 0.019* |
|  |  |  |  | (0.006) |  |  | (0.005) |  |  | (0.007) |  |  | (0.007) |
| E | Post |  |  |  |  |  |  |  |  |  |  |  |  |
|  |  |  |  |  |  |  |  |  |  |  |  |  |  |
|  | Post*Rurality |  |  |  |  |  |  |  |  |  |  |  |  |
|  |  |  |  |  |  |  |  |  |  |  |  |  |  |
| F | Post |  |  |  |  |  |  |  |  |  |  |  |  |
|  |  |  |  |  |  |  |  |  |  |  |  |  |  |
|  | Post*Rurality |  |  |  |  |  |  |  |  |  |  |  |  |
|  |  |  |  |  |  |  |  |  |  |  |  |  |  |

Abbreviations: ACO: accountable care organization. DID: difference in differences. HCC: hierarchical condition category.

**Notes**: The variable *Post* was defined to represent an interaction between ACO participation and time of implementation. Generalized estimating equations (GEE) with logit link function were estimated with population averages for the association between clinic ACO affiliation and changes in the likelihood of the outcome variables, marginal effects and standard errors were reported. All regression models were adjusted for factors including gender, race, age group, insurance type, and number of HCCs (coefficients are available upon request to the authors). Each panel represents a set of individual regressions for each of the 8 outcome variables. Heteroskedasticity robust standard errors were estimated. Inference * p<0.05, ** p<0.01, *** p<0.001. Bonferroni correction was used.

**Panel A**: Repeating the same results as in **Figure 1 and Appendix Table 2**.

**Panel B**: Accounting for four other HCC variables based on all occupational and environmental medicine (OEM) diagnoses with history: HCC institutional score, HCC new enrollee score, HCC special needs plan new enrollee score, and HCC community score.

**Panel C**: Restrict sample to people aged 65 years and older.

**Panel D**: Restricting sample to one-year reporting periods.

**Panel E**: Restricting the range of hemoglobin A1c to 4%-15% for measures, including Blood Sugar (A1c) Poor Control (>9%) and Blood Sugar (A1c) Control (<8%).

**Panel F**: Not restricting the sample to patients with a diabetes indicator based on all OEM diagnoses with history for measure of Tobacco User and Tobacco Cessation Advice.
